# HIV Antigen Incorporation within Adenovirus Hexon Hypervariable 2 for a Novel HIV Vaccine Approach

**DOI:** 10.1371/journal.pone.0011815

**Published:** 2010-07-27

**Authors:** Qiana L. Matthews, Aiman Fatima, Yizhe Tang, Brian A. Perry, Yuko Tsuruta, Svetlana Komarova, Laura Timares, Chunxia Zhao, Natalia Makarova, Anton V. Borovjagin, Phoebe L. Stewart, Hongju Wu, Jerry L. Blackwell, David T. Curiel

**Affiliations:** 1 Division of Human Gene Therapy, Departments of Medicine, Pathology, Surgery, Obstetrics and Gynecology, and the Gene Therapy Center, University of Alabama at Birmingham, Birmingham, Alabama, United States of America; 2 Center for AIDS Research, University of Alabama at Birmingham, Birmingham, Alabama, United States of America; 3 Vision Science Graduate Program, University of Alabama at Birmingham, Birmingham, Alabama, United States of America; 4 Davidson College, Davidson, North Carolina, United States of America; 5 Department of Dermatology, University of Alabama at Birmingham, Birmingham, Alabama, United States of America; 6 Emory University, Atlanta, Georgia, United States of America; 7 Institute of Oral Health Research, University of Alabama at Birmingham, School of Dentistry, Birmingham, Alabama, United States of America; 8 Vanderbilt University Medical Center, Nashville, Tennessee, United States of America; Tsinghua University, China

## Abstract

Adenoviral (Ad) vectors have been used for a variety of vaccine applications including cancer and infectious diseases. Traditionally, Ad-based vaccines are designed to express antigens through transgene expression of a given antigen. However, in some cases these conventional Ad-based vaccines have had sub-optimal clinical results. These sub-optimal results are attributed in part to pre-existing Ad serotype 5 (Ad5) immunity. In order to circumvent the need for antigen expression via transgene incorporation, the “antigen capsid-incorporation” strategy has been developed and used for Ad-based vaccine development in the context of a few diseases. This strategy embodies the incorporation of antigenic peptides within the capsid structure of viral vectors. The major capsid protein hexon has been utilized for these capsid incorporation strategies due to hexon's natural role in the generation of anti-Ad immune response and its numerical representation within the Ad virion. Using this strategy, we have developed the means to incorporate heterologous peptide epitopes specifically within the major surface-exposed domains of the Ad capsid protein hexon. Our study herein focuses on generation of multivalent vaccine vectors presenting HIV antigens within the Ad capsid protein hexon, as well as expressing an HIV antigen as a transgene. These novel vectors utilize HVR2 as an incorporation site for a twenty-four amino acid region of the HIV membrane proximal ectodomain region (MPER), derived from HIV glycoprotein gp41 (gp41). Our study herein illustrates that our multivalent anti-HIV vectors elicit a cellular anti-HIV response. Furthermore, vaccinations with these vectors, which present HIV antigens at HVR2, elicit a HIV epitope-specific humoral immune response.

## Introduction

Adenoviral (Ad) vectors have been used for a variety of vaccine applications including cancer and infectious diseases [1]–[4]. Ad vectors have been utilized as vaccine vectors because of several attributes. This broad utility profile has derived from several key attributes: (a) the viral genome is readily manipulated allowing derivation of recombinant viruses; (b) replication-defective Ads can be derived and propagated easily in complementing cell lines making production of large scale vaccines feasible; (c) Ads infect a broad range of target cells [5], [6]; (d) they possess a large gene delivery payload of up to 8kb; and (e) the vector can achieve unparalleled levels of *in vivo* gene transfer with high levels of induced transgene expression [6], [7]. Traditionally, Ad-based vaccines have been designed to express antigens through transgene expression of a given antigen [8]. However, in some cases these conventional Ad-based vaccines have had sub-optimal clinical results. These sub-optimal results are attributed in part to pre-existing Ad serotype 5 (Ad5) immunity. 50–90% of the adult population has pre-existing immunity (PEI) to Ad5 and therefore, if an individual is vaccinated with an Ad vector for therapeutic purposes there maybe limited transgene/antigen expression in that individual [9]–[13]. In this regard, the “antigen capsid-incorporation” strategy has been developed to circumvent drawbacks associated with conventional transgene expression of antigen within Ad. This strategy embodies the incorporation of antigenic peptides within the capsid structure of viral vectors. This antigen capsid-incorporated strategy has been used for Ad-based vaccines in the context of many diseases [4], [14]–[18]. One of the first instances whereby the antigen capsid-incorporation strategy was used was in research performed by Crompton in 1994. Crompton and colleagues inserted an eight amino acid sequence of the VP1 capsid protein of poliovirus type 3 into two regions of the adenovirus serotype 2 hexon. One of the chimeric vectors produced from this methodology grew well in tissue culture and antiserum raised against the Ad with the polio insert specifically recognized the VP1 capsid of polio type 3.

Using this antigen capsid-incorporation strategy, we have developed the means to incorporate heterologous peptide epitopes specifically within the major surface-exposed domains of the Ad capsid protein hexon. The major capsid protein hexon has been utilized for these antigen capsid incorporation strategies due to hexon's natural role in the generation of anti-Ad immune response and its numerical representation within the Ad virion [4], [14], [15], [17]–[21]. Of note, our previous work has shown that we can incorporate small heterologous peptides into Ad hexon hypervariable regions (HVRs) without perturbing viral viability and other biological characteristics [19]. Published studies have focused on antigen and/or epitope incorporations at HVR5 or single site antigen/epitope incorporation at fiber or protein IX (pIX) [22]. In this regard, antigenic epitopes including linker sequences ranging in size from nine to forty-five amino acids have been incorporated within the Ad5 hexon region or Ad2 hexon region. These epitope incorporations include epitopes derived from polio, *Pseudomonas aeruginosa*, B. *anthracis*, and HIV as well as model epitopes [4], [15], [16], [18]. Based on our ability to manipulate both HVR2 and HVR5 sites, recently we sought to explore the relative merits of antigen incorporation within these two hexon locales. In order to accomplish this we compared the flexibility and capacity of HVR2 and HVR5, respectively. We genetically incorporated identical model epitopes of increasing size within HVR2 or HVR5 of the Ad5 hexon. Our previous study demonstrated that hexon-incorporated model antigens elicit a range of immune responses depending on antigen placement or antigen size at either the HVR2 or HVR5 locales. This study confirmed that HVR2 could be a potential incorporation site for antigens of considerable size and that vaccination with vectors that display incorporations at HVR2 is feasible for vaccine development [16].

Our study herein focuses on the creation of multivalent vaccine vectors presenting HIV antigens in the context of the Ad capsid protein hexon, as well as expressing HIV antigens as a transgene. Specifically, these novel vectors utilize the hexon HVR2 as an incorporation site for a twenty-four amino acid region (EKNEKELLELDKWASLWNWFDITN) of the HIV membrane proximal ectodomain region (MPER), derived from HIV glycoprotein gp41 (gp41). In addition, to presenting MPER within HVR2 these vectors express HIV-specific protein Gag from the Ad5 Δ*E1* region. Our study herein illustrates that our multivalent anti-HIV vectors elicit a humoral and cellular anti-HIV response. Furthermore, vaccinations with these vectors, which present model antigens at HVR2, elicit a HIV epitope-specific humoral immune response.

## Materials and Methods

### Antibodies

For these studies HIV-1 gp41 monoclonal antibody (2F5), cat# 1475 was used. The following reagent was obtained through the NIH AIDS Research and Reference Reagent Program, Division of AIDS, NIAID, NIH: HIV-1 gp41 Monoclonal Antibody (2F5) was generated by Dr. Hermann Katinger. The human monoclonal antibody to HIV-1 gp41 is specific for ELDKWA epitope [23]–[25]. Goat anti-human horse radish peroxidase (HRP) antibody was purchased from Southern Biotech. (Birmingham, AL).

### Cell culture

Human embryonic kidney cells (HEK293) were obtained from and cultured in the medium recommended by the American Type Culture Collection (Manassas, VA). All cell lines were incubated at 37°C and 5% CO_2_ under humidified conditions.

### Recombinant adenovirus construction

In order to generate recombinant adenoviruses with the MPER insertions within the hexon regions, fragments of DNA corresponding to MPER, were generated by PCR from templates provided by Integrated DNA technologies. In our manuscript, the MPER sequence corresponds to EKNEKELLELDKWASLWNWFDITN from HIV gp41. This fragment was subcloned into the BamHI site in the previously described HVR2-His_6_/pH5S plasmids [19]. To create Ad5 vectors containing HIV epitopes in the HVRs of hexon, these resulting plasmids were digested with EcoRI and PmeI. These resulting fragments containing the homologous recombination regions and the hexon genes were purified, then recombined with a SwaI-digested Ad5 backbone vector that lacks the hexon gene, pAd5/ΔH5 [26]. These recombination reactions were performed in *Escherichia coli* BJ5183 (Stratagene, La Jolla, CA). The resultant clone was designated as Ad5/HVR2-MPER-L15; in addition, there was another virus construct with this hexon modification, which contained the HIV Gag gene in the Δ*E1* region: Ad5/HVR2-MPER-L15 (Gag). To create Ad5 vectors containing HIV Gag, the cytomegalovirus (CMV)-Gag construct was subcloned into an Ad Δ*E1* shuttle plasmid. The resulting plasmid was digested with PmeI restriction enzyme. The resulting fragment containing the homologous recombination region at the Ad5 Δ*E1* region was then recombined with the *E1* deleted Ad5 backbone. AdCMVEnv was made as previously described [27], This vector expresses the HIV 89.6 envelope (Env) gene under the control of the CMV promoter.

### Virus rescue and preparation

To rescue viruses the constructed plasmids were digested with PacI and two µg DNA were transfected (Lipofectamine 2000 Reagent, Invitrogen, Carlsbad, CA) into the Ad-*E1*-expressing HEK293 cells. Following plaque formation, they were processed for large-scale propagation in HEK293 cells. Viruses were purified by double cesium chloride ultracentrifugation and dialyzed against phosphate-buffered saline containing 10% glycerol. Viruses were stored at −80°C until use. Final aliquots of virus were analyzed for physical titer using absorbance at 260 nm. The infectious viral titer (IFU) per ml was determined by tissue culture infectious dose (TCID_50_) assay. The TCID_50_ titer was calculated by using KARBER statistical method: *T* TCID_50_ titer  = 10×10^1 + d(*S* − 0.5)^/ml, in which d is the log 10 of the dilution and *S* is the sum of ratios from the first dilution. Modifications of the hexon gene was confirmed by PCR analysis with the primers 5′HVR2 (sense), CTCACGTATTTGGGCAGGCGCC and 3'HVR5 (antisense), GGCATGTAAGAAATATG AGTGTCTGGG, which anneal up and downstream of the site of the insertion within the hexon open reading frame.

### Western blot analysis

To analyze Gag expression, in brief, 1×10^6^ HEK293 cells were infected with various vectors at 100 IFU per cell. Cell lysates were collected after 24 hours and subjected to four freeze-thaw cycles to obtain crude cell lysate. 10 µg of protein was boiled in Laemmli sample buffer for 10 minutes and resolved on 4 to 15% sodium dodecyl sulfate-polyacrylamide gel. The proteins were transferred to polyvinylidene fluoride membrane and staining was performed with Gag antibody (1∶1,000) (Affi-anti-HIV matrix IgY, Genway Biotech, Inc.), followed by secondary staining with HRP-linked goat-chicken IgY (1∶2,000) (Aves Lab, Inc). The proteins were detected on the polyvinylidene fluoride membrane by staining with 3′3′-diaminobenzidine tablets (Sigma-Aldrich, St. Louis, MO).

In brief, to analyze MPER presentation on selected vectors, 10^10^ viral particle (VP) were boiled in Laemmli sample buffer for 10 minutes and resolved on 4 to 15% sodium dodecyl sulfate-polyacrylamide gel. The proteins were transferred to polyvinylidene fluoride membrane and staining was performed with HIV-1 gp41 monoclonal antibody (2F5) (1∶1,000). Followed by secondary staining with HRP-conjugated goat anti-human antibody (1∶2,000). The proteins were detected on the polyvinylidene fluoride membrane by staining with 3′3′-diaminobenzidine tablets (Sigma-Aldrich, St. Louis, MO).

### Whole virus ELISA and sera ELISA

The enzyme-linked immunosorbent assay (ELISA) was performed essentially as described previously [28]. In order to determine if the MPER peptide was surface exposed on the Ad virion whole virus ELISA was performed. Briefly, different amounts of viruses ranging from 4×10^6^ to 9×10^9^ VPs were immobilized on 96-well plate (Nunc Maxisorp, Rochester, NY) by overnight incubation in 100 µl of 100 mM carbonate buffer (pH 9.5) per well at 4°C. After washing with 0.05% Tween 20 in Phosphate-buffered saline (PBS) and blocking with blocking solution (2% bovine serum albumin and 0.05% Tween 20 in PBS), the immobilized viruses were incubated with anti-HIV 2F5 monoclonal antibody (cat# 1475) for 2 hr at room temperature (RT) followed by incubation with an AP-conjugated goat anti-human antibody. Colormetric reaction was performed with *p*-nitrophenyl phosphate (Sigma-Aldrich, St. Louis, MO) as recommended by the manufacturer, and optical density at 405 nm (OD_405nm_) was determined with a microplate reader (Molecular Devices).

For the anti-MPER response ELISA plates (Nunc Maxisorp, Rochester, NY) were coated with 10 µM of the MPER peptide (GenScript Co, Piscataway, NJ) in 100 µl of 50 mM carbonate (pH 9.6) per well, according to the method we described previously [29]. Plates were washed and then blocked with 3% BSA/PBS. After washing, 60 µl of 1∶20 diluted sera was added. After incubation for at least 2 hr at RT, the plates were extensively washed and blocked with 3% BSA/PBS. The plates were then washed with HRP-conjugated goat anti-human antibody (Southern Biotech, Birmingham, AL). ELISAs were developed with TMB substrate. In order to determine MPER isotype-specific reactivity, ELISA plates were coated with 10 µM of the MPER peptide in 100 µl of 50 mM carbonate (pH 9.6) per well, according to the method we described previously [29]. Plates were washed and then blocked with 3% BSA/PBS. After washing, 60 µl of 1∶20 diluted sera was added. After incubation for at least 2 hr at RT, the plates were extensively washed and blocked with 3% BSA/PBS. Isotype-specific mouse antibody (Sigma-Aldrich, St. Louis, MO) was then bound to ELISA plates. Plates were then washed and HRP-conjugated anti-mouse antibody (Dako Denmak, Denmark). ELISAs were developed with TMB substrate (Sigma-Aldrich, St. Louis, MO). OD_450_nm was measured on an Emax microplate reader.

### Growth kinetics

Growth kinetics of Ad vectors were obtained essentially as described previously [26]. HEK293 cells were plated in 6-well plates at the density of 3×10^5^ cells per well 24 h before infection. The cells were infected with Ads at 5 VPs/cell in 500 µl growth medium containing 2% FBS. 1.5 ml more growth medium containing 10% FBS was then added into each well after 2 h incubation at 37°C in 5% CO_2_ humidified incubator. The infected cells were monitored and harvested with medium at various time points post-infection until complete CPE was formed. The collected cells together with the medium were lysed by four freeze–thaw cycles, and subjected to centrifugation at 3000 ×*g* for 30 min at 4°C for cell debris removal. The total viruses in each well were determined by multiplying TCID_50_ titer with the total volume of the supernatant, and plotted as growth curves.

### Thermostability

Heat inactivation assay was performed essentially as described previously [19], [26], [30], [31]. Briefly, viruses were incubated at 45°C for 0, 5, 10, 20 or 40 min in either PBS (without Ca^2+^ and Mg^2+^) or growth medium containing 2% FBS. Then their infectious titers were re-determined by standard TCID_50_ method (AdEasy vector system, Qbiogene Inc., Carlsbad, CA). One day before analysis 10^4^ 293 cells plus 100 µl of growth medium with 2% Fetal Bovine Serum (FBS) were added in 96-well flat bottom plates. Eight serial dilutions of the virus ranging from 10^−3^ to 10^−10^ or 10^−6^ to 10^−13^ were made in medium containing 2% FBS depending on the virus, and 100 µl of each dilution was added into 96-well plates, one row for each dilution. After incubation for 10 days at 37°C in 5% CO_2_ humidified incubator the plates were examined for cytopathic effect (CPE) under microscope. Observable CPE containing wells were counted for each row in order to determine the ratio of positive wells per row in the 96-well plates. Titer was calculated by using TCID_50_.

### Mouse immunization

The following experiment was performed to determine antibody response after immunization with Ad vectors. Female BALB/c (H-2K^d^) mice at 6–8 weeks of age were obtained from the Jackson Laboratory (Bar Harbor, ME). Groups of at least eight mice were analyzed in each experiment or at each time point. Ads were injected into each group of mice: Ad5, Ad5/HVR2-MPER-L15(Gag), Ad5/HVR2-MPER-L15Δ*E1*, AdCMVGag, AdCMVEnv at 1×10^10^ VP per mouse using intramuscular (i.m.) injection. These mice were boosted 14 days after prime with 1×10^10^ VP of the same vector. For CD8 T cell response analysis, blood was collected from mice vaccinated with Ad5/HVR2-MPER-L15(Gag) or AdCMVGag. All animal protocols were approved by the Institutional Animal Care and Use Committee at the University of Alabama at Birmingham.

### MPER peptide synthesis

The antigenic epitope of HIV MPER derived from gp41 was generated in an expression plasmid by (GenScript Co, Piscataway, NJ). This peptide was used as a positive control for the ELISA assays. Peptides were >98% pure as indicated by analytical high-performance liquid chromatography. Peptides were dissolved in 100% DMSO at a concentration of 10 mM and stored at −20°C until use.

### Flow cytometry

For flow cytometric analysis of HIV-1 Gag gene expression, 10^6^ 293AD cells (Qbiogene Inc.) were infected at 10 VP/cell with either AdCMVGag or Ad5/HVR2-MPER-L15(Gag) Ad vectors for 24 hours in the presence of different amounts of human ascites and then cells were permeabilized with Cytofix/Cytoperm (BD Bioscience) at 4°C for 20 min. After washing three times with Perm Wash Buffer (BD Bioscience), cells were incubated with 1∶40 dilution of monoclonal antibody to HIV-1 p24 (AG3.0) at 4°C for 30 min. Cells were washed again and incubated with 1∶10 diluted PE-conjugated anti-mouse IgG (BD Farmingen) at 4°C for 30 min. Cells then were washed and analyzed on FACSCalibur flow cytometer (BD Bioscience). Data were acquired with CellQuest software and analyzed with FlowJo version 8.8.6 software.

### Intracellular flow cytometry staining

Mice were vaccinated and boosted, as described above, peripheral blood Gag-specific CD8 T cells were enumerated by Flow cytometric analyses. Whole blood (100 µl) was collected in heparinized PBS on days 26, 69, and 84 following initial vaccination. Leukocytes were collected using lymphocyte separation medium (Mediatech. Inc. Manassas, VA). Cells were collected and washed twice in 1%BSA, 2mM EDTA in PBS and counted. One to two million lymphocytes were stained with or without Gag Tetramer (APC) (created by Emory University) and a cocktail of CD4-Alexa488 and CD8-PE antibodies (BD Pharmingen, San Jose, California). Cells were washed with 1%BSA, 2mM EDTA in PBS. Cells were fixed with 1% paraformaldehyde and subsequently analyzed for flow cytometry on LSR II cytometer. Analysis of 3,000 CD8 T cells was collected per sample.

### Statistical evaluation

The data are presented as the mean ± the standard error. Statistical analyses were performed with the nonpaired two-tailed Student *t* -test, assuming equal variance. Statistical significance was defined as *P*<0.03.

## Results

### Construction of adenoviral vectors that contain HIV antigen genes

**Figure 1:**
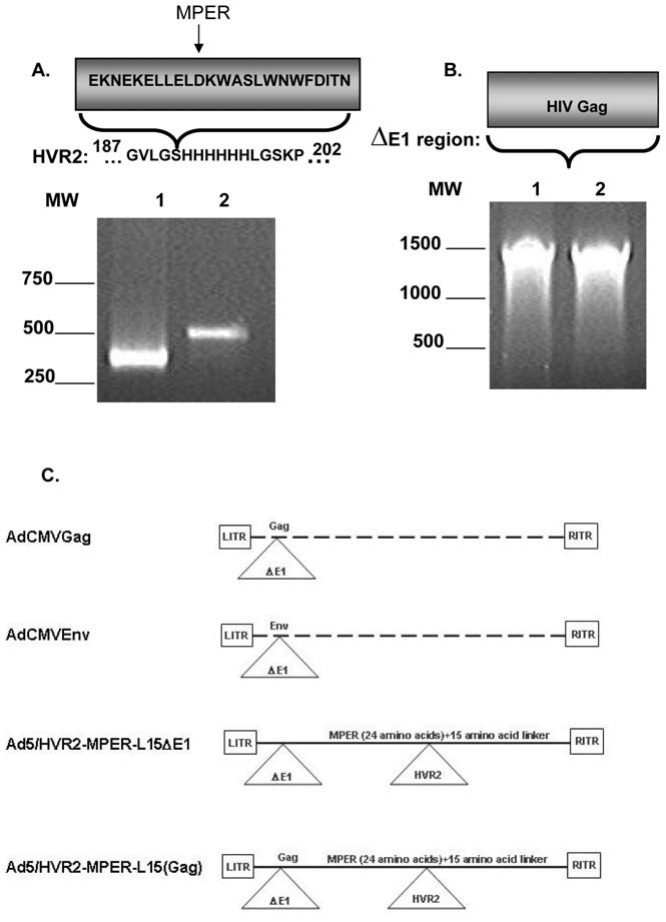
HIV envelope gp41 or Gag genes were genetically incorporated into hexon hypervariable region 2 or adenovirus Δ*E1* region. Rescued viruses were amplified and viral DNA analyzed to confirm stable modification of relevant genes. A) Hexon-specific PCR primers confirmed incorporation of coding regions for MPER epitope inserts at the hexon HVR2 site. Lane 1, AdCMVGag; lane 2, Ad5/HVR2-MPER-L15(Gag). B) Gag-specific primers confirmed the incorporation of coding regions for Gag inserts in the Δ*E1* region. Lane 1, AdCMVGag; lane 2, Ad5/HVR2-MPER-L15(Gag). C) Vectors used for this study are depicted in this figure.

After establishing the technical feasibilities allowing us to place epitopes into HVR2 or HVR5[16], [19], we sought to explore whether we could present HIV antigens within the Ad protein hexon as well as express HIV antigens as a transgene within Ad5. The rationale for choosing a portion of the MPER (EKNEKELLELDKWASLWNWFDITN), derived from gp41 for incorporation into the Ad capsid is based on the fact that the gp41 envelope protein ectodomain is a target of three broadly neutralizing anti-HIV-1 antibodies [32]. Gp41 antigenic function is conformation dependent, therefore we planned to insert a linker sequence onto the MPER core region in order to have the MPER epitope presented on the Ad capsid as close to it's natural conformation as possible [33]. Our vaccine vector design also embodies the incorporation of the HIV Gag gene within the Ad Δ*E1* region. The HIV Gag gene is a major structural protein of the HIV virus. The Gag protein has been frequently used for HIV vaccine schemes [34]. The DNA sequence corresponding to a 24 amino acid region of the MPER plus a 15 amino acid linker was amplified by PCR and then cloned into the hexon HVR2 shuttle vector as previously described [16]. In addition, the HIV Gag gene was subcloned into an Δ*E1* shuttle vector under control of the CMV promoter and transferred into the *E1*-deleted Ad5 genome (Ad5/HVR2-MPER-L15(Gag)) by homologous recombination. In addition, a control vector was generated, which contained an *E1*-deleted Ad5 genome in combination with MPER within the hexon HVR2 region (Ad5/HVR2-MPER-L15Δ*E1*). A control Ad genome expressing the Gag gene under the CMV promoter with no hexon modification (AdCMVGag) was generated in this study as well. The resulting Ad genomes were partially sequenced to confirm that the correct genes were incorporated. Subsequent transfection of HEK293 cells with the resulting recombinant genomes resulted in rescue of the following vectors: AdCMVGag, Ad5/HVR2-MPER-L15Δ*E1,* and Ad5/HVR2-MPER-L15(Gag). In order to further confirm vector identities, hexon and Gag-specific PCR analyses were performed using genomic DNA from the purified virions (Figure 1A-B). With regard to hexon-specific PCR, AdCMVGag was found to have a wild type hexon PCR profile producing ∼450 base pairs (bp) PCR fragment using the hexon-specific primers designed to amplify a regions between HVR2 and HVR5 (Figure 1A, lane 1). A hexon specific PCR, on the Ad5/HVR2-MPER-L15(Gag) construct revealed a 500 bp fragment, suggesting the expected insertion (Figure 1A, lane 2). The addition of ∼50 bp in Figure 1A, lane 2 indicates the incorporation of the MPER-L15 DNA within the hexon region. Both AdCMVGag and Ad5/HVR2-MPER-L15(Gag) were found to be positive in a Gag-specific PCR assay, producing a ∼1500 bp product with Gag-specific PCR primers (Figure 1B, lanes 1–2, respectively). A diagram depicting the vector construction of vectors used in this study is illustrated in Figure 1C.

### Expression and display of HIV antigens within adenoviral vectors

**Figure 2:**
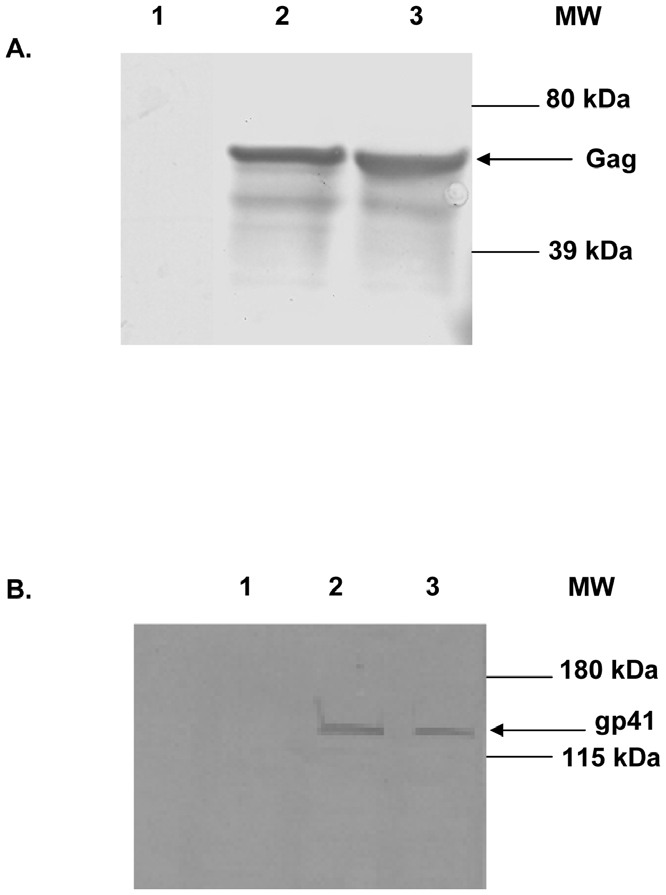
Western blotting confirmed the presence of HIV genes within Ad vectors. A) In the assay, HEK293 cells were infected with various vectors at 100 IFU per cell. Cell lysates were collected after 24 hours and equal protein amounts were separated on a 4 to 15% polyacrylamide gradient SDS-PAGE gel. The proteins were transferred to polyvinylidene fluoride membrane and then stained with Gag antibody. Lane 1, Ad5 negative control; lane 2, Ad5/HVR2-MPER-L15(Gag); lane 3, AdCMVGag. The arrow indicates Gag protein. B) 10^10^ VP of Ad5 (lane 1), Ad5/HVR2-MPER-L15(Gag) (lane 2), and Ad5/HVR2-MPER-L15Δ*E1* (lane 3) were separated on 4 to 15% polyacrylamide gradient SDS-PAGE gel. The proteins were transferred to polyvinylidene fluoride membrane then stained with anti-gp41 antibody. The arrow indicates MPER protein genetically incorporated into the hexon protein.

After successful incorporation of HIV genes we next sought to verify expression of our transgene and capsid incorporation at the protein level by Western blot analysis. HEK293 cells were infected with unmodified Ad5 (as a negative control) or one of the HIV-Gag containing viruses (Ad5/HVR2-MPER-L15(Gag) or AdCMVGag, respectively. Cell lysates were prepared 24 hours post-infection and subjected to Western blot analysis with anti-Gag-specific antibody. Gag protein expression was evidenced by the presence of a ∼55 kDa molecular weight protein band in the lysates from cells infected with either hexon-modified Gag expressing vector Ad5/HVR2-MPER-L15(Gag) (Figure 2-A, lane 2) or the AdCMVGag (Figure 2-A, lane 3). As expected no Gag protein expression was detected in the lysates from cells infected with the unmodified Ad5 virus (Figure 2-A, lane 1).

In order to determine if the hexon-modified vectors were presenting the MPER epitope within the hexon region, purified unmodified Ad5, Ad5/HVR2-MPER-L15(Gag), or Ad5/HVR2-MPER-L15Δ*E1* were subjected to Western blot analysis with anti-gp41 antibody. The MPER protein was detected as a 119 KDa protein band associated with Ad5/HVR2-MPER-L15(Gag) or Ad5/HVR2-MPER-L15Δ*E1* particles, Figure 2-B, lanes 2 and 3, respectively. The size of the 119 KDa band corresponds to the expected size of the Ad5 hexon protein with MPER peptide genetically incorporated into the HVR2 region. There was no MPER protein detected on Ad5 wild type particles (Figure 2-B, lane 1).

### HIV antigens, incorporated within HVR2, are exposed on the virion surface

**Figure 3:**
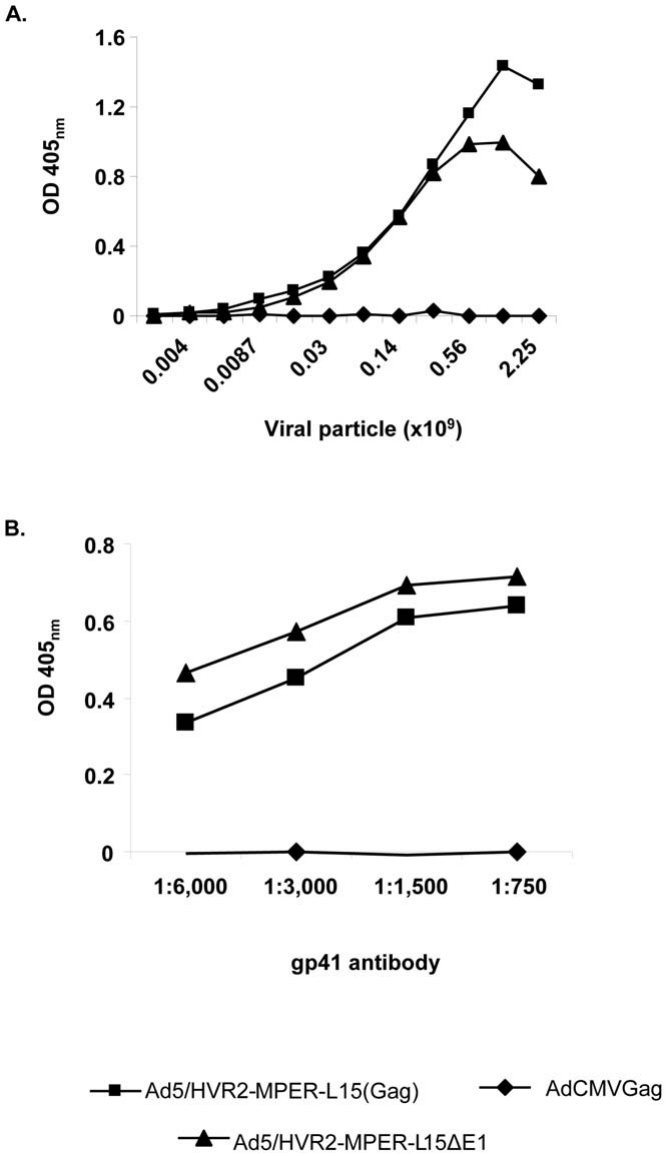
HIV epitopes incorporated in HVR2 are exposed on the virion surface. A) In the assay, varying amounts of AdCMVGag, Ad5/HVR2-MPER24-L15(Gag), and Ad5/HVR2-MPER-L15Δ*E1* were immobilized in the wells of ELISA plates and incubated with anti-gp41 antibody. The binding was detected with an HRP-conjugated secondary antibody. B) In the assay 6×10^8^ VP of either AdCMVGag, Ad5/HVR2-MPER-L15(Gag), and Ad5/HVR2-MPER-L15Δ*E1* were immobilized on an ELISA plate followed by varying dilutions of gp41 antibody (1;6,000; 1∶3,000;1∶1,500; and 1∶750). The binding was detected with an HRP-conjugated secondary antibody.

The foregoing studies validated our ability to derive stable vectors that incorporate MPER within hexon HVR2. Having established the technical feasibilities that allowed us to place large epitopes within hexon's HVR2, we next sought to explore the functional utilities of these modified vectors. To this end, we performed an ELISA assay to verify that the HIV MPER motif in the HVR2 was accessible on the virion surface (Figure 3A). In this assay, varying amounts of purified viruses were immobilized in the wells of an ELISA plate and incubated with anti-gp41 antibody. The results showed significant binding of the anti-gp41 antibody to the Ad5/HVR2-MPER-L15(Gag) and Ad5/HVR2-MPER-L15Δ*E1*, whereas no binding was seen in response to AdCMVGag control. These results indicate that the reactive MPER epitope was properly exposed on the virion surfaces when incorporated within HVR2.

In order to determine the capability of the gp41-specific antibody to bind capsid-incorporated antigen in a dose-dependent manner a dose-response ELISA assay was performed with anti-gp41 antibody. A single concentration of each of the following viruses: AdCMVGag, Ad5/HVR2-MPER-L15(Gag), or Ad5/HVR2-MPER-L15Δ*E1* was applied to ELISA plates, followed by the addition of serial dilutions of gp41 antibody. As predicted, the anti-gp41 antibody bound to Ad5/HVR2-MPER-L15(Gag) and Ad5/HVR2-MPER-L15Δ*E1* in a dose dependent manner (Figure 3B). Our data suggest that the MPER epitope is presented within the hexon in its native conformation as it can be recognized by a monoclonal HIV neutralizing antibody.

### Growth kinetics of Anti-HIV vectors

**Figure 4:**
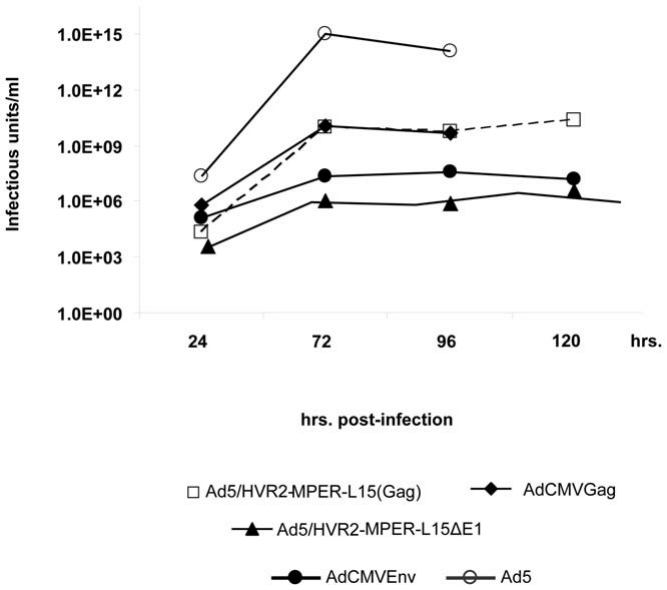
Growth kinetics of modified Ad vectors. HEK293 cells were infected with the vectors respectively, at 5 VP/cell. Cells were harvested with medium at different time points until full CPE was reached and total IFU were determined by TCID_50_. These data were plotted as growth curves.

Previous studies have shown that capsid modifications, as well as some transgene incorporations, can compromise viral growth characteristics. In order to ensure that normal viral growth was not inhibited by epitope and/or transgene incorporation in the viral particle/genome we performed growth kinetic assays on vectors used in this study. To obtain a quantitative understanding of this effect all respective vectors were titered in HEK293 cells until full CPE was achieved in order to determine growth kinetics. The data illustrates that the unmodified Ad5 had the highest titer (∼1×10^14^ IFU/ml) and produced full CPE at 96 hours post-infection. AdCMVGag also produced full CPE at 96-hours post-infection, however, AdCMVGag had a titer, several orders of magnitude lower than that of the Ad5. Ad5/HVR2-MPER-L15(Gag) showed comparable titers to that of AdCMVGag. The control vector expressing full-length envelope protein (AdCMVEnv) reached full CPE after 120 hours post-infection. Ad5/HVR2-MPER-L15Δ*E1* yielded the slowest growth kinetics and relative titer, reaching full CPE at 192 hours post-infection (Figure 4). These growth kinetic patterns were similar to other capsid and transgene modified vectors developed in our laboratory [31], [35].

### Thermostability of Anti-HIV vectors

**Figure 5:**
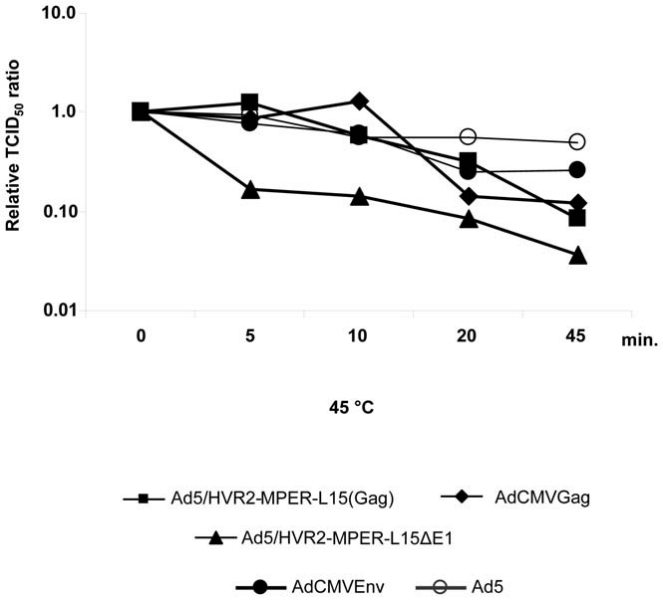
Thermostability of modified Ad vectors. In these assays various viruses were heat inactivated in PBS at 45°C for 0, 5, 10, 20 or 45 minutes. After heat inactivation, the virus were serially diluted and the viral infectious titers were re-determined by TCID_50_ assays.

Previous work has shown that capsid modifications may result in the production of thermoliable vectors. Therefore, we performed thermostability assays with our respective vectors. These particular assays are important due to the complex nature of the incorporated antigens, i.e. MPER and/or Gag. Hexon-modified vectors, as well as controls vectors, were subjected to heating at 45°C for various time points (0,5,10, 20 and 45 minutes). These vectors were then used to infect HEK293 cells. The viral infectious titers were re-determined after heating using TCID_50_ assays. According to the thermostability assay Ad5/HVR2-MPER-L15ΔE1 was the least infectious of all the vectors, showing a dramatic infectivity reduction relative to Ad5 following incubation at 45°C for 20 or 45 minutes (Figure 5). These thermostability results are similar to what we have observed with other transgene-encoding or capsid-modified vectors in our laboratory [31], [35].

### Anti-MPER antibodies produced in mice after vaccination

**Figure 6:**
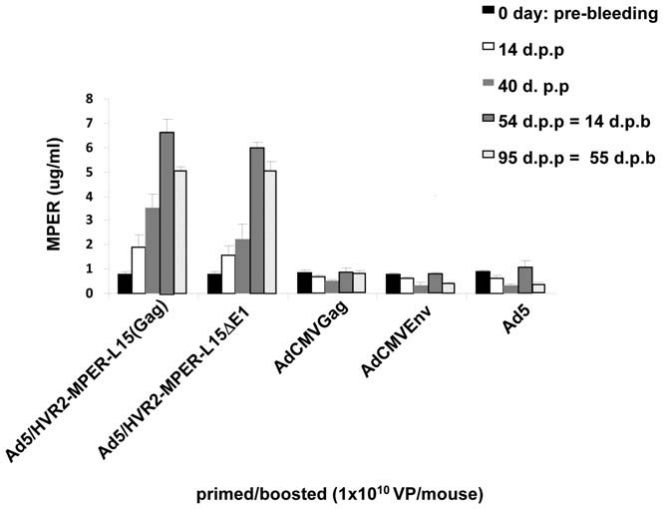
Adenovirus expressing capsid-incorporated HIV antigens elicit an HIV humoral immune response. BALB/c mice (n = 8) were primed and boosted with 10^10^ VP of Ad vectors. Pre-immunization, post-prime, and post-boost sera was collected at various time points for ELISA binding assays. 10 µM of purified MPER (EKNEKELLELDKWASLWNWFDITN) antigenic peptide was bound to ELISA plates. Residual unbound peptide was washed from the plates. The plates were then incubated with immunized mice sera and the binding was detected with HRP conjugated secondary antibody. OD absorbance at 405_nm_ represents MPER antibody levels in sera.

To verify the immunizing potential of these vectors against HIV we next sought to determine if they were capable of eliciting an anti-HIV immune response in mice. Equal numbers (1×10^10^VP) of the vectors were used to immunize BALB/c mice via an intramuscular route. The sera were collected from mice for ELISA assays at various days after priming and boosting. Purified MPER antigenic peptide was bound to ELISA plates. The plates were then incubated with the immunized mice sera. The binding was detected with HRP-conjugated secondary antibody. The amount of anti-MPER antibody in the sera was calculated based on a standard antibody curve dilution. The data demonstrates no binding of the sera from mice immunized with Ad5, AdCMVEnv, or AdCMVGag to the MPER on the plates at any time point. On the contrary, immunization with Ad that contains capsid-incorporated MPER antigen elicits an anti-MPER specific response and allows the formation of MPER-specific antibody in the serum (Figure 6). This immune response is observed at the earliest time point that we observed, which was 14 days post-prime (d.p.p). The peak immune response was observed with the capsid incorporated MPER vectors at 54 d.p.p, which also corresponds to 14 days post-boost (d.p.b.). At 54 d.p.p the determined concentration of gp41 antibody was ∼7 µg/ml. A slight decline of the MPER antibody in the sera at 95 d.p.p was observed in the mice vaccinated with capsid-modified vectors, which corresponded to 55 d.p.b. At 55 d.p.b. MPER antibody levels in the sera was determined to be ∼5 µg/ml. In summary, Ad5/HVR2-MPER-L15Δ*E1* and Ad5/HVR2-MPER-L15(Gag) elicited an anti-gp41 response in vaccinated mice. This MPER-specific response was increased after boosting. It is important to note that the envelope expressed in AdCMVEnv was from the 89.6 HIV variant. The epitope inserted into the Ad5 hexon and the peptide were identical sequences with the 89.6 Env MPER region. The transgene expressed from the Ad5 vector was 89.6 Env (i.e. gp160), which is processed into gp120 and gp41. There were no point mutations within the transgene expressed Env.

### Isotype specific anti-MPER antibodies are produced in mice after vaccination

**Figure 7:**
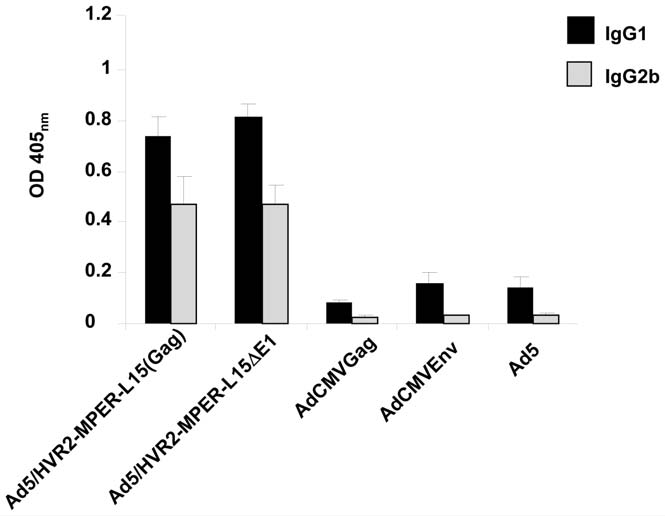
Adenovirus expressing capsid-incorporated HIV antigens elicit an HIV-specific isotype response. BALB/c mice (n = 8) were primed and boosted with 10^10^vp of Ad vectors. Sera was collected 54 d.p.p. and 14 d.p.b. for ELISA isotype binding assays. 10 µM of purified MPER (EKNEKELLELDKWASLWNWFDITN) antigenic peptide was bound to ELISA plates. Residual unbound peptide was washed from the plates. The plates were then incubated with immunized mice sera followed by isotype specific antibodies. The binding was detected with HRP conjugated secondary. OD at 405_nm_ represents of isotype-specific MPER antibody levels in sera.

We next performed experiments to determine the quantitative aspects of the isotype-specific humoral immune responses that were generated in response to the anti-HIV immunization vectors. The sera were collected from mice for ELISA assays at 54 d.p.p and 14 d.p.b., as described previously. Purified MPER antigenic peptide was bound to the plate. The plates were then incubated with immunized mouse sera and the binding was detected with HRP conjugated secondary antibody. Ad5/HVR2-MPER-L15Δ*E1* and Ad5/HVR2-MPER-L15(Gag) vectors produced an isotype-specific anti-MPER humoral response in vaccinated mice; whereas there was no isotype-specific response seen in mice vaccinated with Ad5, AdCMVGag or AdCMVEnv. The presence of isotypes IgG2b and IgG1 corresponds to Th1 and Th2 activation, respectively (Figure 7). T cell activation is important for host protection against foreign antigens.

### Ad5 neutralization of hexon-modified vectors

**Figure 8:**
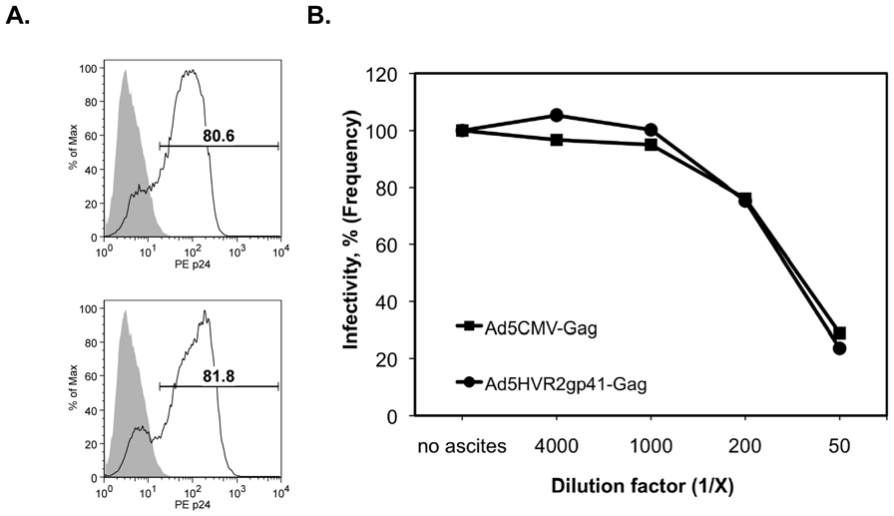
Comparison of infectivity between unmodified and hexon-modified Ad5 vectors. A) 293AD cells were infected at 10 MOI with either AdCMVGag (top panel) or Ad5/HVR2-MPER-L15(Gag) (bottom panel) adenoviral vectors for 24 hours and then Gag expression was measured by flow cytometry and analyzed with FlowJo version 8.8.6 software. The numbers indicate the percentage of cells positive for Gag expression. B) The experiment was repeated as in A (one of three representative experiments is shown) in the presense or absense of serially-diluted neutralizing ascites. The percentage (%) of infectivity was calculated by normalizing Gag expression to the infectivity in the absense of neutralizing ascites.

The incorporation of the HIV-1 epitope into the hexon protein of Ad5 may interfere with the viral infectivity and/or reduce recognition by anti-Ad5 neutralizing antibodies (NAb). We therefore compared infectivity of Ad5/HVR2-MPER-L15(Gag) to that of the original AdCMVGag virus in the presence or absence of anti-Ad5 NAb found in human ascites that have been described previously [36]. In the absence of ascites, the frequency of cells expressing Gag after infection with AdCMVGag or Ad5/HVR2-MPER-L15(Gag) was 81.8% and 80.6% respectively, indicating that the incorporation of HIV-1 epitope into the hexon protein of the Ad5 vector did not reduce virus infectivity (Figure 8A). Compared to the unmodified Ad5 vector, the presence of the HIV-1 epitope did not alter neutralization by anti-Ad5 NAb present in the human ascites (Figure 8B). Therefore the HIV-1 epitope did not facilitate escape from pre-existing anti-adenoviral humoral immune response, probably due to the polyclonal nature of the ascites NAbs.

### Gag Positive T cells produced and boosted in mice after vaccination with hexon-modified vectors

**Figure 9:**
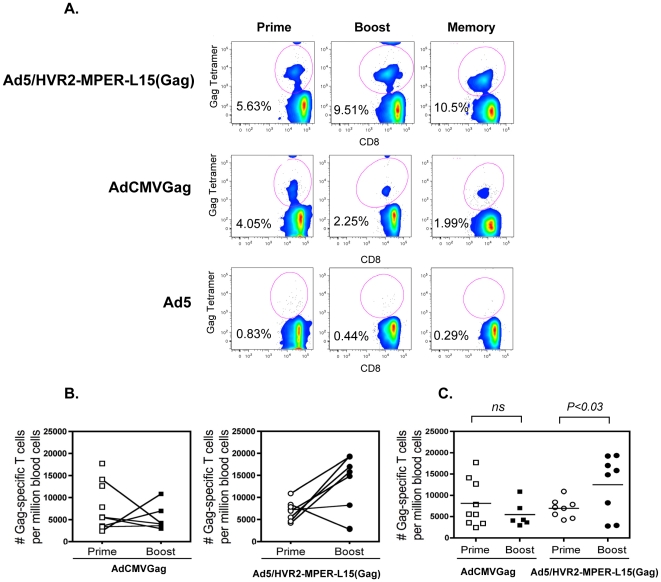
Capsid-modified vectors can induce a greater number of Gag-specific CD8 T cells and memory T cells than wild type vectors. Cohorts of BALB/c mice (n = 8) were immunized (prime) by injection of 10^10^ vp i.m. with of one of the following Ad vectors: AdCMVGag, Ad5/HVR2-MPER-L15(Gag), and Ad5/HVR2-MPER-L15Δ*E1*. Gag-specific T cells were detected in the peripheral blood of mice 2 weeks following the initial vaccination, and given a boost immunization in the same manner on day 40. Peripheral blood Gag-specific CD8 T cells were enumerated 26 d.p.p, 69 d.p.p, and 84 d.p.b. A) Flow cytometric analyses bivariate pseudocolor plots are shown for a single mouse from each group for each time point. B) The percent and total number of Gag-specific T cells per million lymphocytes are shown for each mouse and their level of T cells linked for prime and boost time points. C) Paired scatter plots show significant differences in the percent and number of Gag-specific CD8 T cells induced by either MPER-modified or AdCMVGag gene encoding vaccines. Statistical significance was determined by student's t-test, (two-tailed) P<0.03.

The previous experiments rely on the immune recognition of capsid proteins and do not address the capacity of these modified vectors to transduce cells and direct expression of the delivered antigen-encoding transgenes in vivo. To test the capacity of our hexon-modified vectors to deliver and induce expression of the HIV Gag transgene, we measured the expansion of Gag-specific CD8 T-cells at various consecutive time points following vaccine priming and boosting, and later measured persistence of memory T cells. Equal amounts of viral particles were used to immunize mice. Blood was collected from mice on 21 d.p.p. and 26 d.p.b. T-cells were subjected to Gag tetramer staining. The results in Figure 9 illustrate that after mouse immunization with Ad5/HVR2-MPER-L15(Gag) (5.63%) or AdCMVGag (4.05%) we observed comparable Gag responses between these immunization groups, this is an acceptable result in that the data shows that there was not a loss of Gag response due to the incorporation of MPER epitope within the hexon capsid. Mouse immunization with Ad5 vector resulted in background levels of T-cell activation (0.83%). In addition, Ad5/HVR2-MPER-L15(Gag) (9.51%) vector allowed boosting of the Gag transgene expression as compared to the AdCMVGag vector (2.25%) (Figure 9), these results were statistically significant at p<0.03. Intracellular IFNγ staining in tetramer positive CD8 T cells was observed for all groups (data not shown), confirming the functional integrity of the expanded HIV Gag-specific CD8 T cells.

## Discussion

We have developed novel Ad vectors that have the potential to optimize Ad vaccine approaches. This strategy involves inserting antigenic epitopes into HVR2 or HVR5 regions of the Ad capsid protein, hexon, to stimulate epitope-specific antibody responses following vaccination. This method offers the ability to compare a range of identical epitopes incorporated within HVRs for antigenic optimization. Our current study is the first of its kind to genetically incorporate HIV antigen within the Ad5 hexon HVR2 alone or in combination with genomic incorporation of a transgene for HIV antigen expression. In this study we successfully incorporated the HIV MPER in the Ad5 HVR2 region as evidenced by PCR, Western blot analysis, and the purified, whole-virus ELISAs (Figures 1,2, and 3).

We believe that further characterization of the gp41 binding antibodies is necessary; we performed additional ELISA experiments using purified gp41 protein and sera from our vaccinated mice. We were able to observe a slight binding of whole gp41 protein to the sera of mice vaccinated with Ad vector presenting MPER within the hexon capsid (data not shown). We speculate that this binding would have been increased if we were able to obtain an identical purified gp41 protein as that presented with our capsid-incorporated MPER sequence. This was not possible due to the divergency of HIV protein structures and reagents available. We also performed an ELISA assay with HIV pseudo particles and sera from our vaccinated mice (data not shown). The results were similar to that seen with purified gp41 protein. We speculate that it is difficult to detect binding of gp41 to the HIV particle due to the need of further optimization of this assay and/or the limited amount of gp41 molecules available on a single HIV virion (∼10–30 Env (gp120 and gp41) molecules per virion).

It is more than likely that the MPER epitope inserted into the Ad hexon is not presenting in the same conformation as it would be presented within the context of an HIV virion, therefore there are advantages and disadvantages to the antigen capsid-incorporation approach. The advantages of this approach include the fact that it is obviously much easier to insert a small peptide from HIV into hexon as compared to inserting a correctly folded version of the full HIV protein into hexon (which would likely be impossible). However, despite the fact that the MPER presentation might be different with respect to Ad capsid incorporation compared to that of MPER expressed on the HIV virion, we were able to detect the presence of this epitope by the means of human HIV monoclonal antibody 2F5, which recognizes 14 amino acids within MPER (Figures 2B and 3). Whereas, we were not able to detect the presence of MPER with the use of human HIV monoclonal antibody 4E10, which binds a 6 amino acid linear epitope immediately C-terminal to the 2F5 epitope and contained with MPER (data not shown). These observations suggest that the MPER conformation is similar but not identical to that presented by the HIV virion. One advantage of the antigen capsid-incorporation approach is that the non-glycosylated MPER may be more immunogenic because of the absence of glycan-shielding. With the hexon/peptide presentation approach we should be able to address additional interesting questions, e.g. are there any glycosylation sites within or around the MPER region that affect immunogenicity. We plan to rigorously examine the ability of the peptide presentation approach to produce neutralizing antibodies, perhaps in a guinea pig or rabbit system.

When the MPER was incorporated into HVR2 in combination with transgene expression, we observed growth kinetics (Figure 4) and thermostability (Figure 5) changes similar to that of other capsid-modified vectors generated in other studies [31], [35]. Although, there appears to be substantial growth kinetic and thermostability differences between Ad5/HVR2-MPER-L15(Gag) and Ad5/HVR2-MPER-L15Δ*E1* (Figures 4 and 5); there appears to be no significant difference between cellular Gag immune response generated from vaccination with Ad5/HVR2-MPER-L15(Gag) and Ad5/HVR2-MPER-L15Δ*E1* (Figures 6 and 7). Most importantly, vaccination with hexon-modified vectors in this study resulted in a humoral anti-HIV response. This is noteworthy, because HVR2 has not been fully explored for ‘antigen capsid-incorporation’ strategies. Anti-MPER humoral responses could be boosted after homologous Ad vector administration. At 40 d.p.p the MPER response in mice, vaccinated with hexon-modified vectors was approximately from 6 to 7 times higher than when using AdCMVGag, AdCMVEnv, or Ad5 vector. This is a critical finding of our study. It appears that vaccination with AdCMVEnv does not yield an antibody response. This finding could be explained in various ways: (1) since the HIV Env protein is a precursor protein that is catalyzed by protease to give the final product gp41 and gp120, it is possible that the production of gp120 is masking the antibody recognition of gp41 [37]; (2) this finding might indicate that the antigen capsid-incorporation strategy is superior to antigen transgene expression with regards to generation an in vivo immune response to MPER and/or other antigens [14]. In order to confirm the validity of the results obtained from all vaccination groups, a separate set of ELISA experiments were performed whereby sera from all vaccinated mice were bound to unmodified Ad vectors. All of the sera from the vaccinated groups bound unmodified wild type Ad5 equally well, indicating equal vaccination by Ad5, AdCMVGag, AdCMVEnv, Ad5/HVR2-MPER-L15(Gag) and Ad5/HVR2-MPER-L15Δ*E1* (data not shown). In addition, anti-MPER IgG1 and IgG2b isotype-specific responses were observed in mice vaccinated with the MPER capsid-modified vectors (Figure 7). Similar results were seen with IgG2a (data not shown), however; no MPER IgA-specific response was observed (data not shown). We did not expect to find much of the MPER IgA-specific immune response after vaccination with these vectors due to the route of vector administration. However, we plan to further investigate IgA-specific response in the context of targeted hexon-modified vectors in combination with alternative routes of administration. From a humoral immunity standpoint, IgA2 subclasses are reduced in saliva upon HIV infection, and total secretory IgA levels are reduced at later disease stages. Salivary IgA can be neutralizing to HIV-1 and HIV-2, and can block epithelial transmigration. In this instance, salivary IgA can be functional against HIV [38]–[40].

Figure 8, compares the infectivity of Ad5/HVR2-MPER-L15(Gag) to that of the AdCMVGag vector in the presence or absence anti-Ad5 NAb found in human ascites. Both vectors were equally neutralized in the presence of human ascites fluid. We expected that Ad5/HVR2-MPER-L15(Gag) would have been neutralized to a lesser degree than AdCMVGag in the presence of human ascites. However, since this was not the case, we speculate that the polyclonal anti-Ad5 antibodies found in human ascites fluid is not masked by the addition of a single epitope incorporated within the hexon region. We also speculate that we might have observed different results with this assay if we had used monoclonal antibody, targeted to the hexon region where the insert was incorporated. If a monoclonal antibody was used in the presence of ascites fluid, Ad5/HVR2-MPER-L15(Gag) might escape neutralization largely as compared to AdCMVGag. However, this possibility was not explored further at this time because it does not diminish the clinical benefit of these vectors. Importantly, the Ad5/HVR2-MPER-L15(Gag) vector allowed boosting of Gag transgene production, as compared to the wild type Gag expressing vector (Figure 9), this result was statistically significant at p<0.03. This is an important finding for two reasons: (1) it indicates the potential for a second administration of the same vector without the diminished production of transgene and (2) it demonstrates that the incorporation of HIV epitopes within HVR2 does not have a detrimental effect on in vivo transgene production (Figure 9). We speculate that the MPER-modified vector allows boosting compared to AdCMVGag, possibly because the Ad5/HVR2-MPER-L15(Gag) Ad elicits less anti-Ad5 immune response. It is possible that the MPER epitope reduced the immunogenicity of the Ad5 vector. It is also possible that the anti-MPER antibody fraction was not neutralizing with regards to the hexon-modified Ad5 boost. Similar results were seen with experiments performed by Abe and colleagues, their studies support the concept that modified hexon thwarts Ad5 neutralizing antibodies and promotes cellular immune responses [20].

There have been a range of studies that have now investigated the viral antigen capid-incorporation strategy as a viable means to improve vaccination in many disease or infection contexts [4], [14], [15], [17], [18], [21]. Due to the controversial HIV STEP trial and the continuing HIV epidemic, some groups have employed the antigen capsid-incorporation strategy to make strides against HIV/AIDS by attempting to make a safe and effective HIV vaccine [41], [42]. The main vector systems that have been utilized to derive a HIV vaccine include human rhinovirus (HRV) and Ad-based vector systems [43]. With respect to the HRV system, researchers have constructed HRV:HIV chimeras in an effort to stimulate immunity against HIV-1 [44]. Furthermore or along these lines, in an effort to develop HIV vaccines researchers within this same group have generated combinatorial libraries of HRV capid-incorporated HIV-1 gp41 epitope. Their results indicate that they have been successful in eliciting antibodies whose activity can mimic the 2F5 effect [45].

Commercial and clinical Ad development for HIV vaccines have progressed preferentially more than vector systems such as HRV because, the plasticity of Ad generally exceeds current rhinovirus systems. For example, because it is a relatively small RNA virus, the rhinovirus platform can display an array limited to 60 copies of a single HIV-1 epitope [44]. In comparison, the Ad hexon-incorporation display platform could present an array of 720 HIV-1 epitope copies and the pIX incorporation display platform could potentially present an array of 240 HIV-1 epitope copies. Another significant difference between the two platforms is in the number of locales that have been successfully utilized for heterologous epitopes insertion. In this regard, to our knowledge, the human rhinovirus 14-based platform utilizes a single-epitope insertion site in the major immunogenic portion of the viral VP2 loop 2 [46], [47]. In contrast, the Ad vector platform, could potentially allow incorporation of the HIV-1 MPER epitope into four structurally distinct locales, including: hexon, (HVR2 and HVR5) [20], protein IX, penton base and/or fiber. Lastly, in contrast to the rhinovirus that lacks this capacity, the Ad platform has sufficient coding capacity allowing for HIV-1 transgene expression in combination with presenting the same or a different antigen on the viral capsid surface. Despite some of the noted limitations with the rhinovirus platform, one of its attractive features has been the previously mentioned development of mutagenic libraries of human rhinovirus 14 chimeras that each display randomized residues representing HIV-1 V3 or MPER epitopes [48], [49]. In this regard, it is worth mentioning that our lab has previously developed a similar approach for screening and identifying heterologous amino acid sequence insertions which have been incorporated into the adenovirus fiber knob region using a modified phage display approach [50]-[52]. Overall, through extensive and ongoing vector development by both our group and others, the Ad platform offers a dynamic level of plasticity that translates into the advantage of multiple vaccine development [20].

Taken together, our current study demonstrates that utilization of the HVR2 for HIV MPER epitope incorporation, in combination with HIV Gag transgene incorporation within the Ad genome. Our previous work describes the precise optimal antigen size and configuration for HVR5 capsid incorporation. Based on our ability to manipulate Ad5 capsid proteins as well as internal gene loci, we will be able to establish novel vectors that allow for the production of multivalent vectors that may act as a vaccine ‘cocktail’. These vectors with capsid-incorporated antigens will have the potential to vaccinate patients against HIV by producing a robust anti-HIV humoral and T cell response. Our data illustrates that there is a diverse immunological response/profile of vaccination with capsid-modified vectors compared to that of vector containing wild type hexon. This fact provides the rationale that a heterologous prime boost strategy with our hexon-modified vector followed by the wild type hexon vector could potentially achieve an even greater anti-HIV immune response. In addition, these vectors may have great potential to circumvent the drawbacks seen in the general population related to Ad5 PEI. Our future plans include transitioning these vectors to a ‘gutless’ [53]–[57] system in order to determine the duration and quality of HIV antibody production. In combination with the ‘gutless system’ we plan to decrease Ad5 PEI through the use of chemical conjugates such as Polyethylene Glycol [58], [59] and/or Ad vector chimeras.
